# The value of magnetic resonance imaging in the preoperative diagnosis of tibial plateau fractures: a systematic literature review

**DOI:** 10.1007/s00068-022-02127-2

**Published:** 2022-10-28

**Authors:** Gregoire Thürig, Alexander Korthaus, Karl-Heinz Frosch, Matthias Krause

**Affiliations:** 1 Department of Trauma and Orthopaedic Surgery, University Medical Center Hamburg-Eppendorf, 20251 Hamburg, Germany; 2 Department of Orthopaedic Surgery and Traumatology, Fribourg Hospital Cantonal, Chem. Des Pensionnats 2-6, 1752 Villars-Sur-Glâne, Switzerland; 3 Department of Trauma Surgery, Orthopaedics and Sports Traumatology, BG Hospital Hamburg, 21033 Hamburg, Germany

**Keywords:** Tibial plateau fracture, Knee MRI, Ligament knee injury, Knee CT, Preoperative diagnosis

## Abstract

**Purpose:**

The outcome of a tibial plateau fracture (TPF) depends on the fracture reduction achieved and the extent of soft-tissue lesions, including lesions in the ligaments, cartilage, and menisci. Sub-optimal treatment can result in poor knee function and osteoarthritis. Preoperative planning is primarily based on conventional X-ray and computed tomography (CT), which are unsuitable for diagnosing soft-tissue lesions. Magnetic resonance imaging (MRI) is not routinely performed. To date, no literature exists that clearly states the indications for preoperative MRI. This systematic review aimed to determine the frequency of soft-tissue lesions in TPFs, the association between fracture type and soft-tissue lesions, and the types of cases for which MRI is indicated.

**Methods:**

A systematic review of the literature was based on articles located in PubMed/MEDLINE and the Cochrane Central Register of Controlled Trials (CENTRAL), supplemented by searching the included articles’ reference lists and the ePublication lists of leading orthopedic and trauma journals.

**Results:**

A total of 1138 studies were retrieved. Of these, 18 met the eligibility criteria and included a total of 877 patients. The proportion of total soft-tissue lesions was 93.0%. The proportions of soft-tissue lesions were as follows: medial collateral ligament 20.7%, lateral collateral ligament 22.9%, anterior cruciate ligament 36.8%, posterior cruciate ligament 14.8%, lateral meniscus 48.9%, and medial meniscus 24.5%. A weak association was found between increasing frequency of LCL and ACL lesions and an increase in fracture type according to Schatzker's classification. No standard algorithm for MRI scans of TPFs was found.

**Conclusion:**

At least one ligament or meniscal lesion is present in 93.0% of TPF cases. More studies with higher levels of evidence are needed to find out in which particular cases MRI adds value. However, MRI is recommended, at least in young patients and cases of high-energy trauma.

## Introduction

Tibial plateau fractures (TPFs) are rare, representing 1.0% of all fractures, and are associated with an increased risk of post-traumatic osteoarthritis (OA) [1]. Cruciate ligament injuries, meniscal tears, and TPFs are the greatest risk factors for developing OA [2]. In addition to the quality of the reduction of the articular surface [3], the outcome for knee function also depends on the extent of the soft-tissue injury, including injury to the ligaments, cartilage, and menisci. Sub-optimal treatment for torn ligaments can result in poor knee function [4] and persistent instability, which may require reoperation [5]. Therefore, treatment of soft-tissue lesions should be included in future guidelines to improve the outcome of TPFs.

The initial diagnostic modalities for a suspected TPF are usually X-ray and computed tomography (CT). Because clinical examination of the ligamentous structures is not always possible in the acute condition due to swelling and patient pain [6], attempts have been made to draw conclusions from fracture morphology in relation to possible soft-tissue injury using conventional X-ray and CT [7, 8]. The resulting observations allow the surgeon to calculate the probability of additional soft-tissue injury, depending on the articular depression and tibial plateau widening, but not a precise indication of which injured structures should be surgically addressed. These methods only sensitize the surgeon to the eventuality of additional internal injuries. However, a clear prediction of injured internal structures could help surgeons to plan operations. For this purpose, magnetic resonance imaging (MRI) is preferred [9], and various authors recommend MRI for preoperative TPF evaluation and surgical planning [10–12].

Fracturoscopy is another strategy that has been suggested as an intraoperative diagnostic tool with the possibility of detecting and treating internal knee damage during treatment. However, no greater benefit in diagnosis or therapy has been shown compared to traditional treatment [13].

The literature is inconsistent regarding the added benefits of preoperative MRI for planning TPF treatment. To better understand this issue, we performed a systematic review that aimed to determine the frequency of soft-tissue lesions in TPF, the association between fracture type and soft-tissue lesions, and the cases for which MRI is indicated.

## Materials and methods

A systematic review was performed following the Preferred Reporting Items for Systematic Reviews and Meta-Analysis (PRISMA) guidelines (PROSPERO: CRD42021244398).

### Search strategy

We searched PubMed/MEDLINE and the Cochrane Central Register of Controlled Trials (CENTRAL) using the Boolean operators OR/AND: ((MRI OR “magnetic resonance imaging” AND (“tibial plateau fracture” OR “proximal tibia fracture”)). The search was supplemented by searching the reference lists of the included articles and the ePublication lists of leading orthopedic and trauma journals, including *Clinical Orthopaedics and Related Research*, *Archives of Orthopaedic and Trauma Surgery*, *Journal of Bone and Joint Surgery* (American and British versions), *Injury*, and *The Knee*. All the studies that evaluated outcomes were selected, and the full text was obtained. Biomechanical, in vitro and in vivo studies, review articles, articles about surgical techniques, case reports, letters to the editor, editorials, and conference abstracts were excluded.

### Data extraction

Two authors (GT and AK) independently reviewed the titles and abstracts of each article identified in the literature search. The full text of each article was obtained and evaluated for eligibility when the eligibility was unclear from the title and abstract. Any disagreements were resolved through a consensus discussion between the two independent reviewers. A third reviewer (MK) was consulted if consensus could not be achieved.

The full text of the selected articles was screened for the following inclusion criteria: (1) a prospective or retrospective study design on MRI prior to TPF surgery; (2) a comparative or correlative study on MRI and conventional X-ray and/or CT for TPF.

Articles were excluded based on the following criteria: (1) an incomplete comparison or correlation of conventional X-ray or CT and MRI; (2) no reported classification of proximal tibial fracture; (3) no tissue lesions described; and (4) studies written in a language other than English. No restrictions were imposed on demographic characteristics, surgical technique, choice of classification, or choice of osteosynthesis material.

**Fig. 1:**
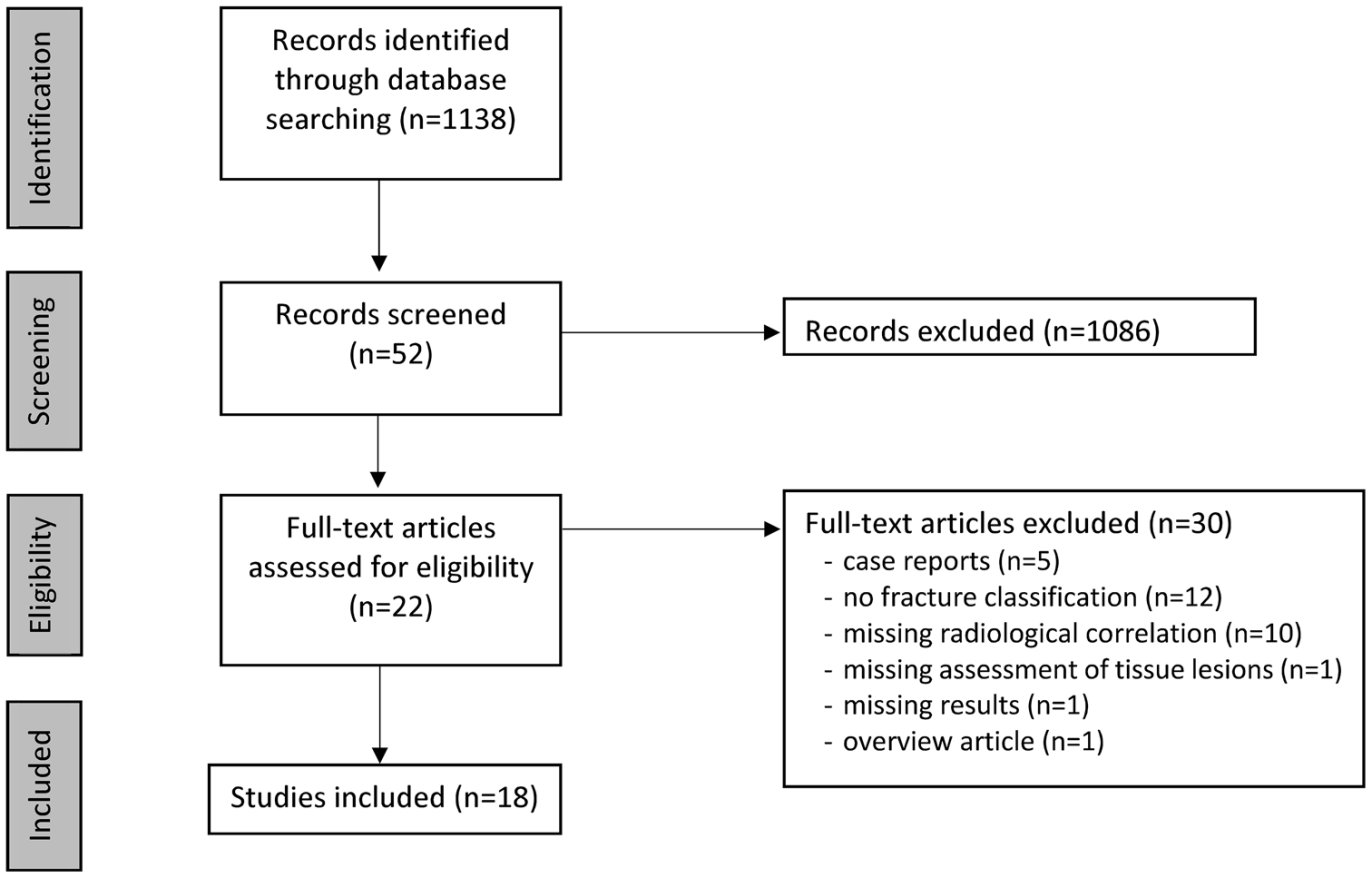
PRISMA flowchart of the included and excluded studies

The initial search produced 1139 articles. After reviewing the abstracts, 22 articles were eligible for initial screening according to the inclusion and exclusion criteria. All 22 articles presented an assessment of TPF with conventional X-ray, CT, and MRI, and were therefore included in the preliminary analysis. Four articles were excluded: two were excluded for not reporting whether every patient had a conventional X-ray or CT scan, one was excluded for only reporting the fracture classification without evaluating tissue lesions, and one was excluded for not reporting the results. Therefore, 18 studies were included in this systematic review [7, 8, 10–12, 14–26] (Fig. 1).

### Study selection, inclusion and exclusion criteria, and quality assessment and evaluation

The Centre for Evidence-Based Medicine (OCEBM)’s modification for orthopedic literature [27] was used by two reviewers (GT and AK) to independently assess the included studies and assign a level of evidence from I to IV (Table 1). The methodological quality of the included studies was assessed using the methodological index for nonrandomized studies (MINORS) checklist [28] (Table 1). In accordance with the MINORS checklist, the non-comparative studies were evaluated using eight items, each rated from 0 to 2 points, comprising the study aim, consecutive patients, a prospective design, appropriate endpoints, unbiased assessment, follow-up, the dropout rate, and the sample size calculation. Four additional items, including an adequate control group, contemporary groups, baseline group equivalence, and adequate statistical analysis, were used for the comparative studies. The maximum possible scores were 16 and 24 for the non-comparative and comparative studies, respectively. The reviewers (GT, AK) discussed any discrepancies regarding study quality, and the senior author (MK) was consulted about any disagreements.

**Table 1:** MINOR checklist

Study	Year	Level of evidence	Type of study	Clearly stated aim	Inclusion of consecutive patients	Prospective collection of data	Endpoints appropriate to the aim of the study	Unbiased assessment of the study endpoint	Followup period appropriate to the aim of the study	Loss to followup \5%	Prospective calculation of the study size	An adequate control group	Contemporary groups	Baseline equivalence of groups	Adequate statistical analysis	Total score
Barrow [22]	1994	III	Retrospective	2	2	0	2	2	0	0	0	2	2	2	0	10/24
Kode [26]	1994	III	Prospective	2	0	2	1	2	0	0	0	2	2	0	0	7/24
Holt [10]	1995	IV	Prospective	2	2	0	1	1	0	0	0	1	2	2	0	8/24
Colletti [11]	1996	IV	Retrospective	2	0	0	2	0	0	0	0	n/a	n/a	n/a	n/a	4/16
Yacoubian [20]	2002	III	Retrospective	2	0	2	2	2	0	0	0	2	2	2	2	15/24
Gardner [12]	2005	IV	Prospective	2	2	2	2	1	0	2	0	n/a	n/a	n/a	n/a	11/16
Gardner [25]	2006	IV	Retrospective	2	2	0	2	1	0	0	0	n/a	n/a	n/a	n/a	7/16
Mui [14]	2006	III	Retrospective	2	2	0	2	1	0	0	0	2	2	2	2	11/24
Kolb [7]	2008	III	Retrospective	2	0	0	2	1	0	0	0	2	2	2	2	9/24
Mustonen [15]	2008	IV	Retrospective	2	0	0	2	2	0	0	0	n/a	n/a	n/a	n/a	6/16
Stannard [21]	2010	IV	Retrospective	2	0	0	2	2	0	0	0	n/a	n/a	n/a	n/a	6/16
Spiro [8]	2013	IV	Retrospective	2	2	0	2	2	0	0	0	n/a	n/a	n/a	n/a	8/16
Wang [17]	2015	IV	Prospective	2	0	2	2	2	0	0	0	n/a	n/a	n/a	n/a	8/16
Wang [23]	2016	IV	Retrospective	2	0	0	2	2	0	0	0	n/a	n/a	n/a	n/a	6/16
Park [16]	2017	IV	Retrospective	2	0	0	2	0	0	0	0	n/a	n/a	n/a	n/a	4/16
Warner [18]	2018	IV	Retrospective	2	0	2	1	1	1	0	0	n/a	n/a	n/a	n/a	7/16
Choi [24]	2018	III	Retrospective	2	0	0	2	0	0	0	0	2	2	1	2	7/24
Yan [19]	2020	IV	Retrospective	2	0	0	2	1	0	0	0	n/a	n/a	n/a	n/a	5/16

The items were scored 0 (not reported), 1 (reported but inadequate), 2 (reported and adequate)

*n/a* not applicable

### Outcome measures

The patient demographic details, including age, sex, mode of trauma, and the radiological investigation used, were extracted to provide an overview of the population. The type of classification, total lesions, lesions to collateral and cruciate ligaments, lesions to menisci, and lesions by classification were obtained. Information regarding recommendations for additional MRI diagnostics was gathered. The data were synthesized in narrative and tabular formats.

### Data analysis

The data analyses were performed using R statistical software (v4.2.0; R Core Team 2021) with the R function “metaprop” from the R package “meta” (v5.2-0; Schwarzer 2022) and IBM SPSS Statistics version 26 program (IBM Corp., Armonk, NY, USA).

In the cases of normally distributed data, an analysis using the Freeman–Tukey double arcsine transformation was performed. In the cases of skewed distribution, an approach based on the logit transformation using generalized linear mixed models was performed, according to the recommendation of Schwarzer et al. [29].

The proportions and 95% confidence intervals (CIs) were calculated for 7 dichotomous variables: total soft-tissue lesions and medial collateral ligament (MCL), lateral collateral ligament (LCL), anterior cruciate ligament (ACL), posterior cruciate ligament (PCL), and medial and lateral meniscus lesions.

The *I*^2^ statistic was used to evaluate heterogeneity, with *I*^2^ > 50% indicating significant heterogeneity, as was Cochran’s Q *p* value, with a *p* value < 0.05 indicating significant heterogeneity. A random-effects model was used throughout. Egger’s and Begg’s tests were performed for each variable to test for small-study effect. A *p* value < 0.05 denoted statistical significance.

A subgroup analysis to measure the association between the different types according to Schatzker’s classification [30] and the individual soft-tissue lesions was performed using a rank bi-variate correlation (Spearman’s *r*_rb_, with a *p* value < 0.05 indicating statistical significance). The value was calculated according to Cohen [31] (*r*_rb_ ≥ 0.1 to < 0.3 corresponds to a weak effect, *r*_rb_ ≥ 0.3 to < 0.5 corresponds to a moderate effect, and *r*_rb_ ≥ 0.5 corresponds to a strong effect).

## Results

### Methodological quality

Due to the presence of a control group (Table 1), 6/18 studies were Level III, according to the OCEBM criteria. The remaining 12 studies were level IV. The areas of best performance based on the MINORS checklist were the statement of a clear aim and endpoints appropriate to the study’s aim. The areas of worst performance based on the MINORS checklist were the follow-up period, a loss-to-follow-up ratio less than 5.0%, and prospective calculation of the study size. Most studies (14/18) had a retrospective design. The median MINORS score for the non-comparative studies was 6.5 (range 4–11) out of a maximum score of 16, and the median score for the comparative studies was 11 (range 11–16) out of a maximum score of 24 (Table 1).

### Demographics and radiographic modality

A total of 877 patients, with a mean age of 41.82 (14–87) years, suffered 878 TPFs. Of these, 322 patients were female, and 463 were male, while two studies did not provide information on sex [14, 17]. Nine studies [7, 16, 20–26] disclosed the type of trauma mechanism. A mean 61.5% of these 387 fractures were due to high-energy trauma (high-velocity and traffic accidents).

The chosen radiological modalities were conventional X-ray and MRI in four studies [11, 12, 17, 26], CT and MRI in two studies [20, 23], and conventional X-ray, CT, and MRI in five studies [8, 15, 21, 22, 24]. In two studies, conventional X-rays were compared with MRI [10, 14]. Four studies compared CT with MRI [10, 16, 18, 19], and one study compared conventional X-ray with CT and MRI [25] (Table 2).

**Table 2:** Demographic, trauma mechanism and radiographic modality

Study	Year	Number of subjects	Average Age	Sex (female: male)	Trauma mechanism	Investigation modalities (X-ray, CT, MRI)
Barrow [22]	1994	30 (31 fractures)	38 (19–71)	–	–	Biplane linear tomography vs MRI
Kode [26]	1994	22	20–85	07:15	–	CT vs MRI
Holt [10]	1995	21	41 ( 18–81)	08:13	–	X-ray vs MRI
Colletti [11]	1996	29	38 ( 21–77)	10:19	–	X-ray, MRI
Yacoubian [20]	2002	52	42.6 (24–72)	14:38	52 high energy (100%)	X-ray vs CT vs MRI
Gardner [12]	2005	103	46 ( 14–82)	40:63	–	X-ray, MRI
Gardner [25]	2006	62	48 ( 14–82)	–	–	X-ray, MRI
Mui [14]	2006	41	50 (19–83)	18:23	–	CT vs MRI
Kolb [7]	2008	55	52.6 (± 18)	33:22	64% high energy trauma (car 20.6%), motorcycle (17.7%), or kite surfing (8.8%)	CT vs MRI
Mustonen [15]	2008	39	37 (17–76)	18:21	19 traffic (48%); 9 fall (23%); 6 sports (15%); 5 twisting (13%)	CT, MRI
Stannard [21]	2010	103	40.5 (18–83)	33:70	motor vehicle 58 (56%); high energy falls 18 (17%); motorcycle 10 (9%); pedestrian struck by motor vehicle 9 (9%); equestrian 4 (4%); crush injury 3 (3%); airplane accident 1 (1%)	X-ray, MRI
Spiro [8]	2013	54	51.2 (± 18.3)	34:20	–	X-ray, CT, MRI
Wang [17]	2015	54	48.3 (27–69)	19:35	Motor vehicle 22 (41%); fall from height 12 (22%); fall onto the ground 20 (37%)	X-ray, CT, MRI
Wang [23]	2016	25	53.4 ( 23–68)	07:18	–	X-ray, CT, MRI
Park [16]	2017	25	49 (22–68)	08:17	19 traffic (76%); 3 slip down (12%); 2 ski/football (1%); 1 fall from height (1%)	X-ray, CT, MRI
Warner [18]	2018	53	Injury LM 46.7 ± 11.3 (29–60); no injury LM 42.1 ± 10.1 (34–60)	Injury LM 5:18; no injury LM 6:24	Low energy 9 (16%), high energy 14 (26%)	CT vs MRI
Choi [24]	2018	82	54 (13–87)	42:40	43 (54%) low energy, 29 (37%) high energy, 7(9%) unknown	CT, MRI
Yan [19]	2020	27	54.9 (30–71)	20:07	27 high energy (100%)	X-ray, CT, MRI

### Total soft-tissue lesions

Due to skewed distribution, the meta-analysis of single proportions was conducted using the logit transformation [29]. The following information is displayed in detail in Table 3.

**Table 3:** Overview of incidence of the tissue lesions

Study	Number of subjects	Percentage of total tissue lesions and meniscal lesions	MCL (total/complete/partial)	LCL (total/complete/partial)	PLC and popliteus (total/complete/partial)	ACL (total/complete/bony avulsion/partial)	PCL (total/complete/partial)	Lateral meniscus (total)	Medial meniscus (total: type of lesions)	assigned to classification
Barrow [22]	31	–	3 (10%)/–/–	3 (10%)/–/–	–	7 (23%)/–/–/–	0/–/–	10 (32%)	17 (55%)	Schatzker
Kode [26]	22	68% ligament injury 55% meniscal injury	5 (23%)/–/–	3 (14%)/–/–	–	6 (27%)/–/–/–	1 (5%)/–/–	9 (41%)	3 (14%)	–
Holt [10]	21	47.60%*	0/–/–	4 (19%)/–/–	–	2 (10%)/–/–/–	2 (10%)/–/–	1 (5%)	5 (24%)	–
Colletti [11]	29	97%*	16 (55%)/–/–-	10 (34%)/–/–	–	12 (41%)/–/–/–	8 (28%)/–/–	13 (45%)	6 (21%)	Schatzker
Yacoubian [20]	52	–	–	–	–	–	–	–	–	–
Gardner [12]	103	99%*	37 (36%)/–/–	30 (29%)/–/–	70 (68%) /—/ -	59 (57%)/–/–/–	29 (28%)/–/–	94 (91%)	45 (44%)	–
Gardner [25]	62	-	22 (35%)/–/–	11 (18%)/–/–	36 (58%) /—/ -	35 (56%)/–/–/–	15 (24%)/–/–	45 (73%)	23 (37%)	Schatzker II
Mui [14]	41	87.8% total*; 66% ligament injury; 59% meniscal injury	2 (5%)/–/–	2 (5%)/–/–	–	14 (34%)/–/–/–	3 (7%)/–/–	21 (53%)	11 (27%)	–
Kolb [7]	55	78%*	8 (16%)/–/–	26 (47%)/–/–	–	16 (29%)/–/–/–	5 (9%)/–/–	19 (35%)	11 (20%)	–
Mustonen [15]	39	16 (42%) meniscal tear total, 14 (35%) of them unstable tear; unstable evident 10 medial (25%) and 7 lateral (18%); stable 7 (18%) medial and 10 (25%) lateral; complex 9 (12%), bucket handle 2 (3%)	2 of 13 (33%) surgical repair/–/–	3 of 8 (21%) surgical repair/–/–	-	15 of 19 (49%) surgical repair/–/–/–	9 of 12 (31%) surgical repair/–/–	17 (44%)	16 (41%)	-
Stannard [21]	103	50 (49%) meniscal injury; 73 (71%) ligament injury; 55 (53%) multiple ligament injuries	16 (%)/–/–	46 (%)/–/–	–	45 (%)/–/–	41 (%)/–/–	35 (34%)	25 (24%)	Schatzker and AO
Spiro [8]	54	73 (71%) single ligament injury; 55 (53%) multiple ligament injury; 50 (49%) sustained 60 meniscal tears; 10 (10%) both menisci	4 (7.4%)/0/4 (7.4%)	28 (52%)/15 (27.8%)/13 (24.0%)	–	18 (31.5%)/4 (7.4%)/3 (3.7%)/11 (20.4%)	7 (13%)/0/5 (9.3%)/2 (3.7%)	19 (35.2%)	6 (11.1%)	–
Wang [17]	54	60 (73%) single tissue injury; 33 (40%) multiple tissue injuries	5 (9.3%)/–/–	5 (9.3%)/–/–	–	9 (16.7%)	2 (3.7%)/–/–	30 (55.6%): 14 peripheral tear (25.9%); 12 (22.2%) horn tear	7 (13%): 6 (11.1%) peripheral tear; 1 (1.9%) horn tear	Schatzker
Wang [23]	25	100%*	16 (64%)/1 (4%)/15 (60%)	19 (72%) / 3 (12%) / 16(64%)	–	20 (80%)/–/–/–	9 (36%)/–/–	12 (48%)	1 (4%)	Schatzker and AO
Park [16]	25	–	6 (24%)/–/–	0/–/–	–	4 (16%)/–/–/–	4 (16%)/–/–	16 (64%): 10 repairs (40%), 2 partial meniscectomy (5%), 4 nothing (10%)	1 (4%)	–
Warner [18]	53	43.40%*	–	–	–	–	–	23 (43%)	–	Schatzker
Choi [24]	82	–	23 (28%)/–/–	9 (11%)/–/–	–	8 (10%)/–/–/–	2 (2%)/–/–	49 (60%)	32 (39%)	–
Yan [19]	27	100% total*; 100%ligamentary injury; 81% meniscal injury	8 (29.6%)/–/–	14 (51.9%; 3 bony)/–/–	–	25 (92.6%)/2 (7.4%)/–/–	19 (70%)/0/19 (70%)	17 (63%)	12 (44.4%)	Schatzker IV, AO
total	878	93.0% total lesions of 354 patients in 8 studies(*)	173 (20.7%) of 773	213 (22.9%) of 773	–	295 (36.8%) of 773	159 (14.8%) of 773	430 (48.9%) of 826	221 (24.5%) of 773	8 Schatzker 3 AO

**Fig. 2:**
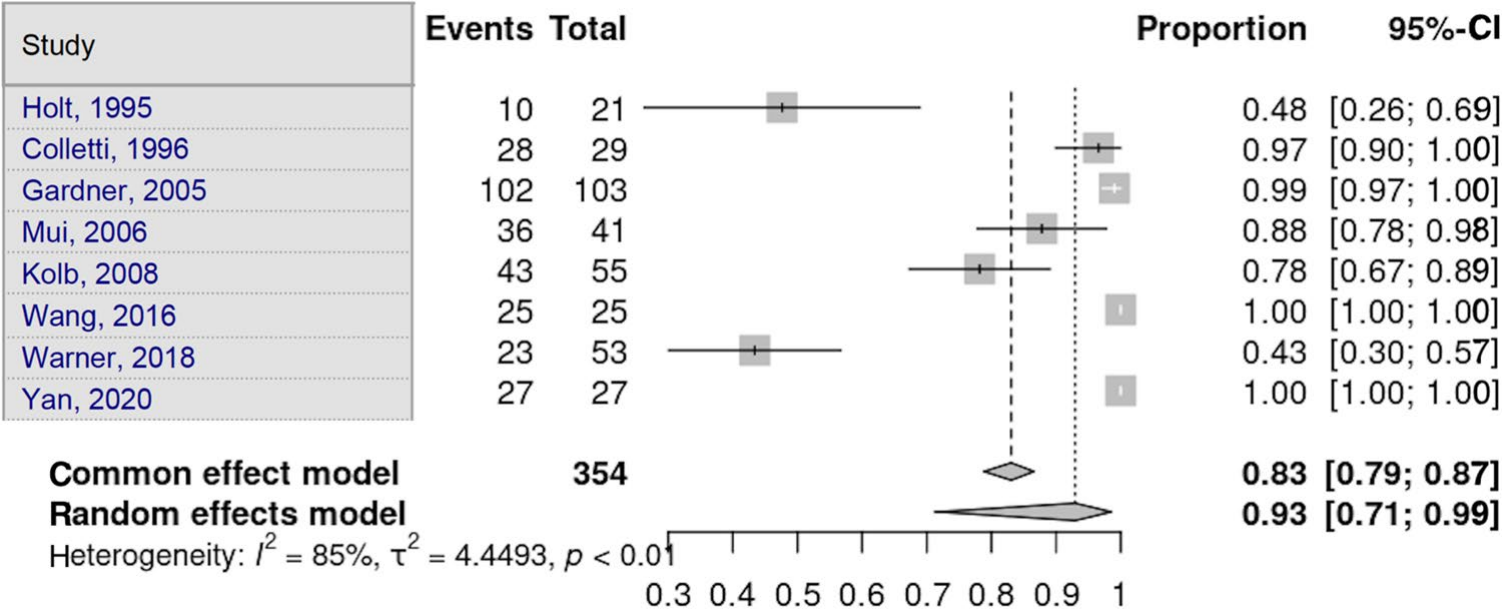
Total tissue lesion

Eight studies disclosed the proportion of patients who suffered at least one soft-tissue lesion in TPF [5–8, 12, 13, 16, 21]. Heterogeneity in the study estimates was assessed using the *I*^2^ statistic (85.3%) and Cochran’s *Q* test (*p* < 0.0001), which showed heterogeneity. The total soft-tissue lesions had an overall proportion of 93.0% (95% CI 71.20–98.59), independent of the fracture type. This was a statistically significant result. Egger’s test and Begg’s test showed no small-study effects (*p* = 0.662 and *p* = 1.000, respectively) (Fig. 2).

**Fig. 3:**
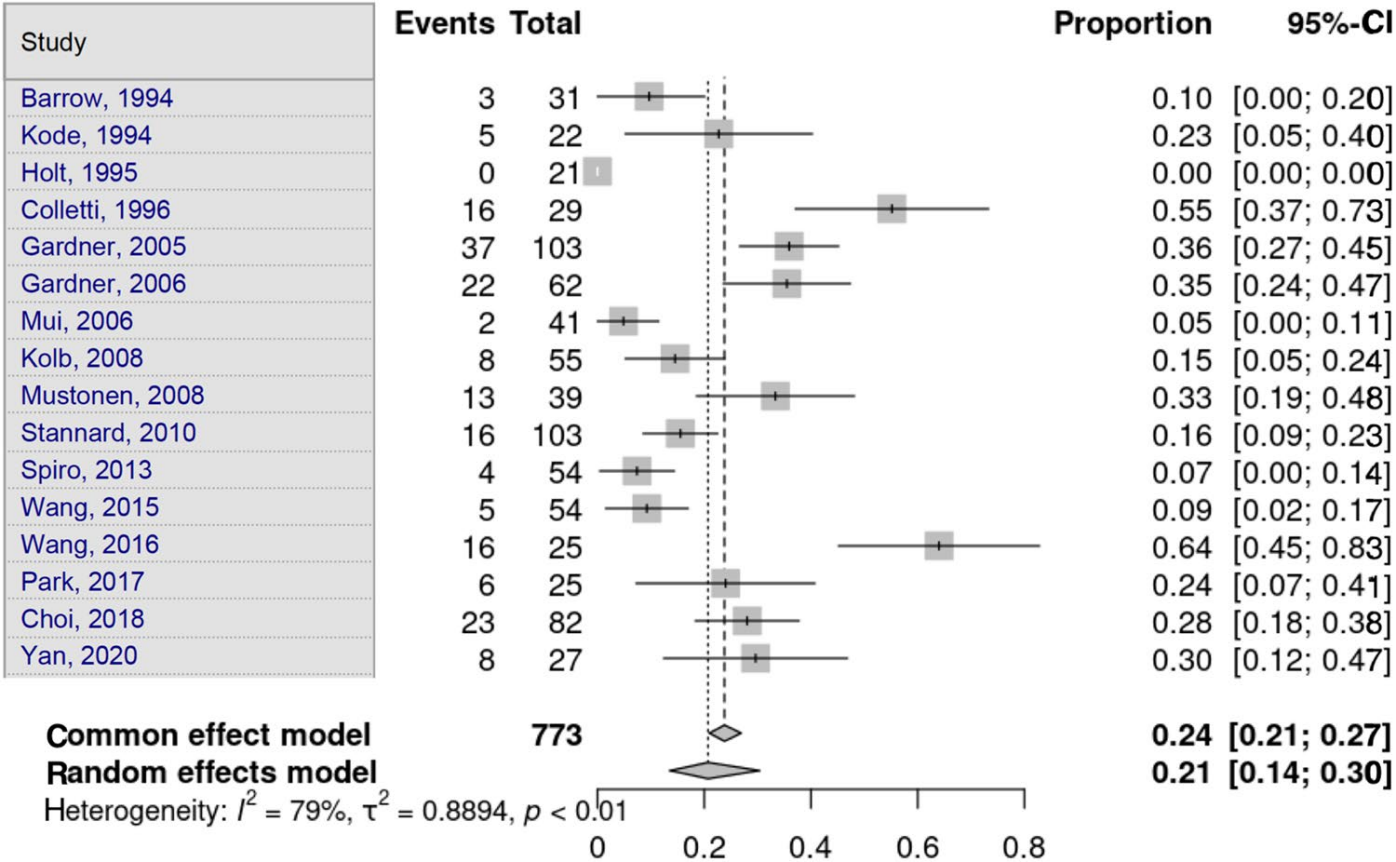
Medial collateral ligament lesion

MCL lesions were assessed by 16 studies. Heterogeneity in the study estimates was assessed using the *I*^2^ statistic (78.8%) and Cochran’s *Q* test (*p* < 0.0001), which showed heterogeneity. The overall proportion was 20.7% (95% CI 13.51–30.42), independent of the fracture type, which was statistically significant. Egger’s test and Begg’s test showed no small-study effects (*p* = 0.969 and *p* = 0.682, respectively) (Fig. 3).

**Fig. 4:**
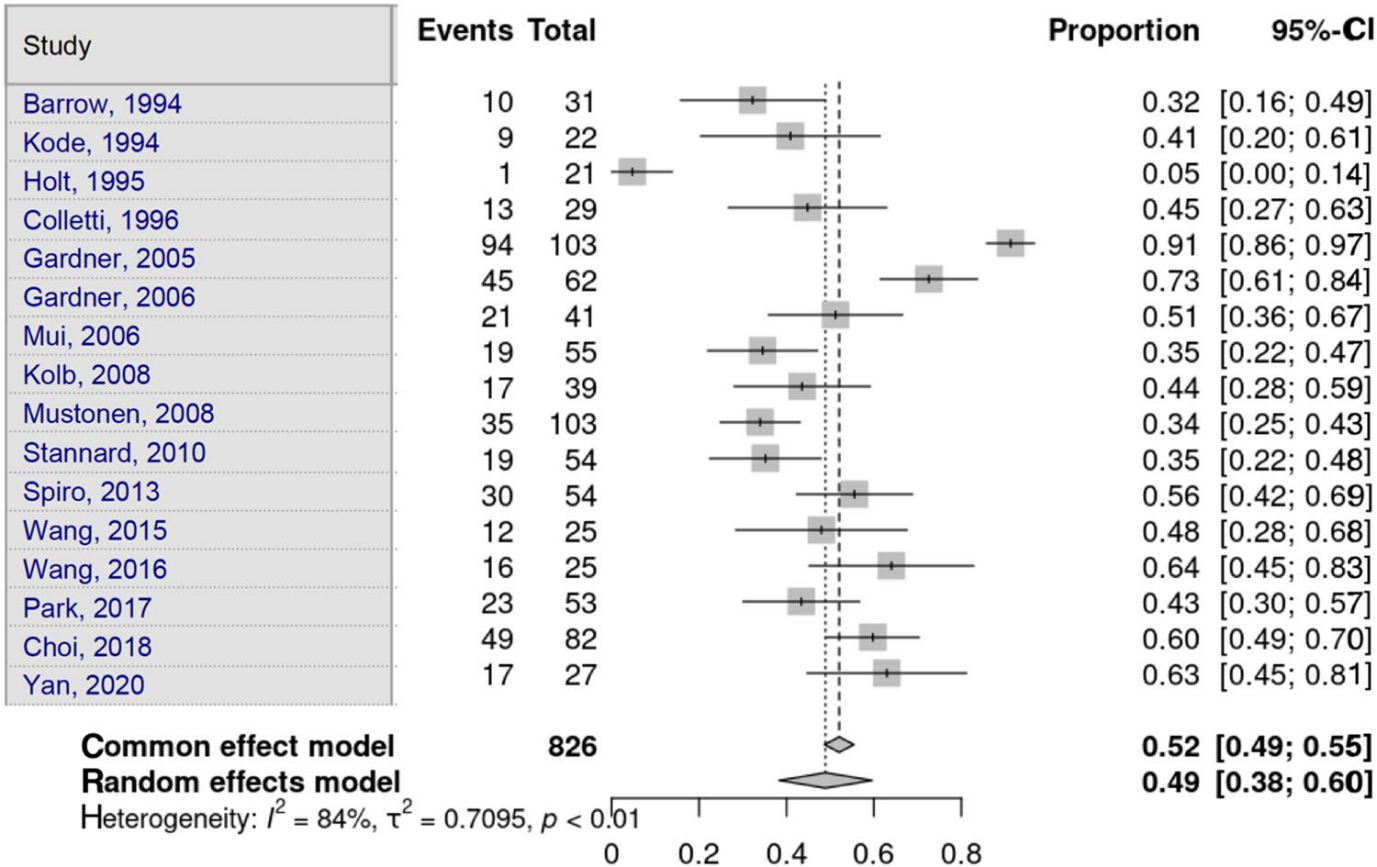
Lateral collateral ligament lesion

LCL lesions were assessed by 16 studies. Heterogeneity in the study estimates was assessed using the *I*^2^ statistic (84.5%) and Cochran’s *Q* test (*p* < 0.0001), which showed heterogeneity. The overall proportion of 22.9% (95% CI 14.01–35.2) was independent of the fracture type and statistically significant. Egger’s test and Begg’s test showed no small-study effects (*p* = 0.670 and *p* = 0.891, respectively) (Fig. 4).

**Fig. 5:**
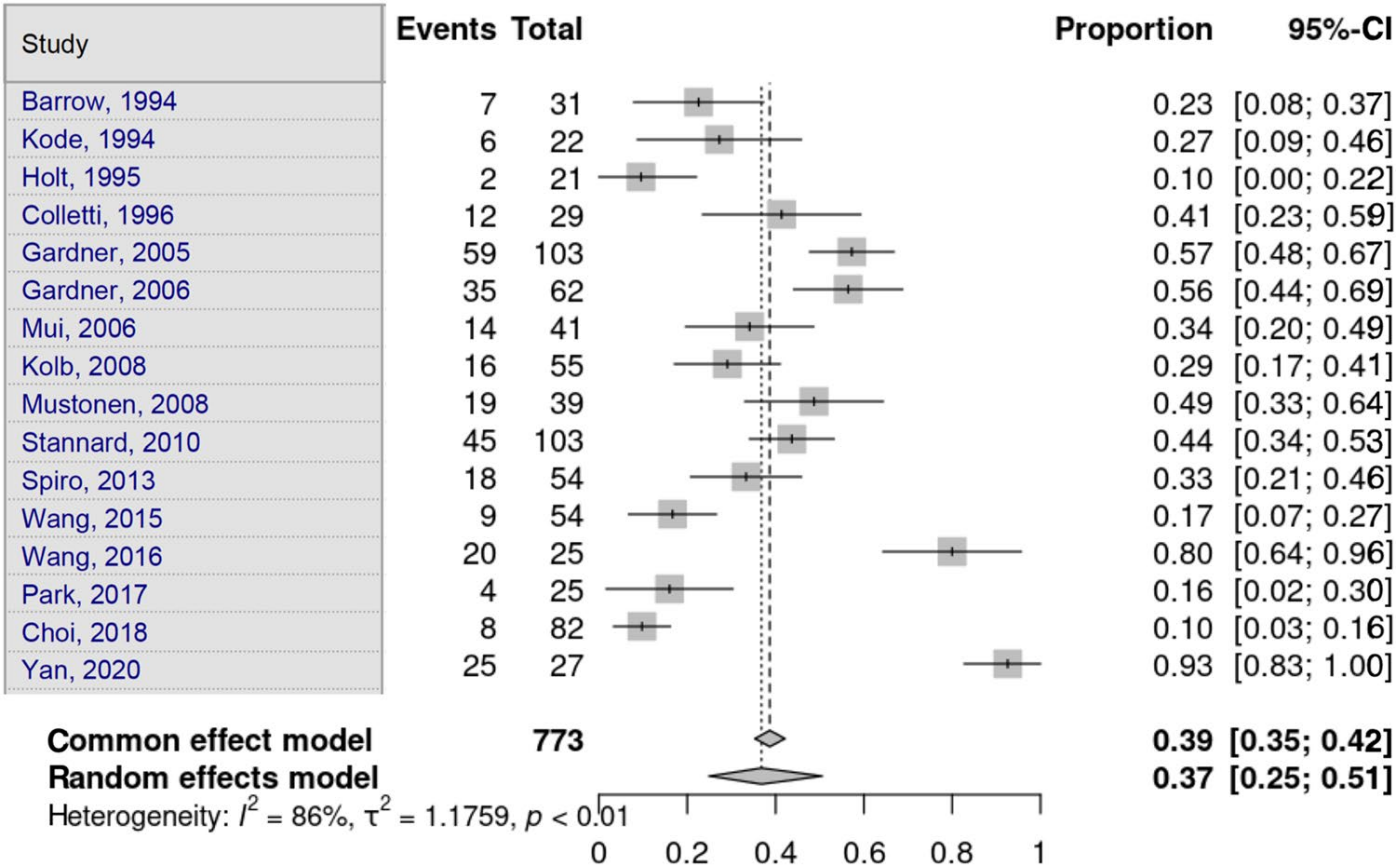
Anterior cruciate ligament lesion

ACL lesions were assessed by 16 studies. Heterogeneity in the study estimates was assessed using the *I*^2^ statistic (85.7%) and Cochran’s *Q* test (*p* < 0.0001), which showed heterogeneity. The overall proportion of 36.8% (95% CI 24.87–50.61) was independent of the fracture type, which was a statistically significant result. Egger’s test and Begg’s test showed no small-study effects (*p* = 0.979 and *p* = 0.750, respectively) (Fig. 5).

**Fig. 6:**
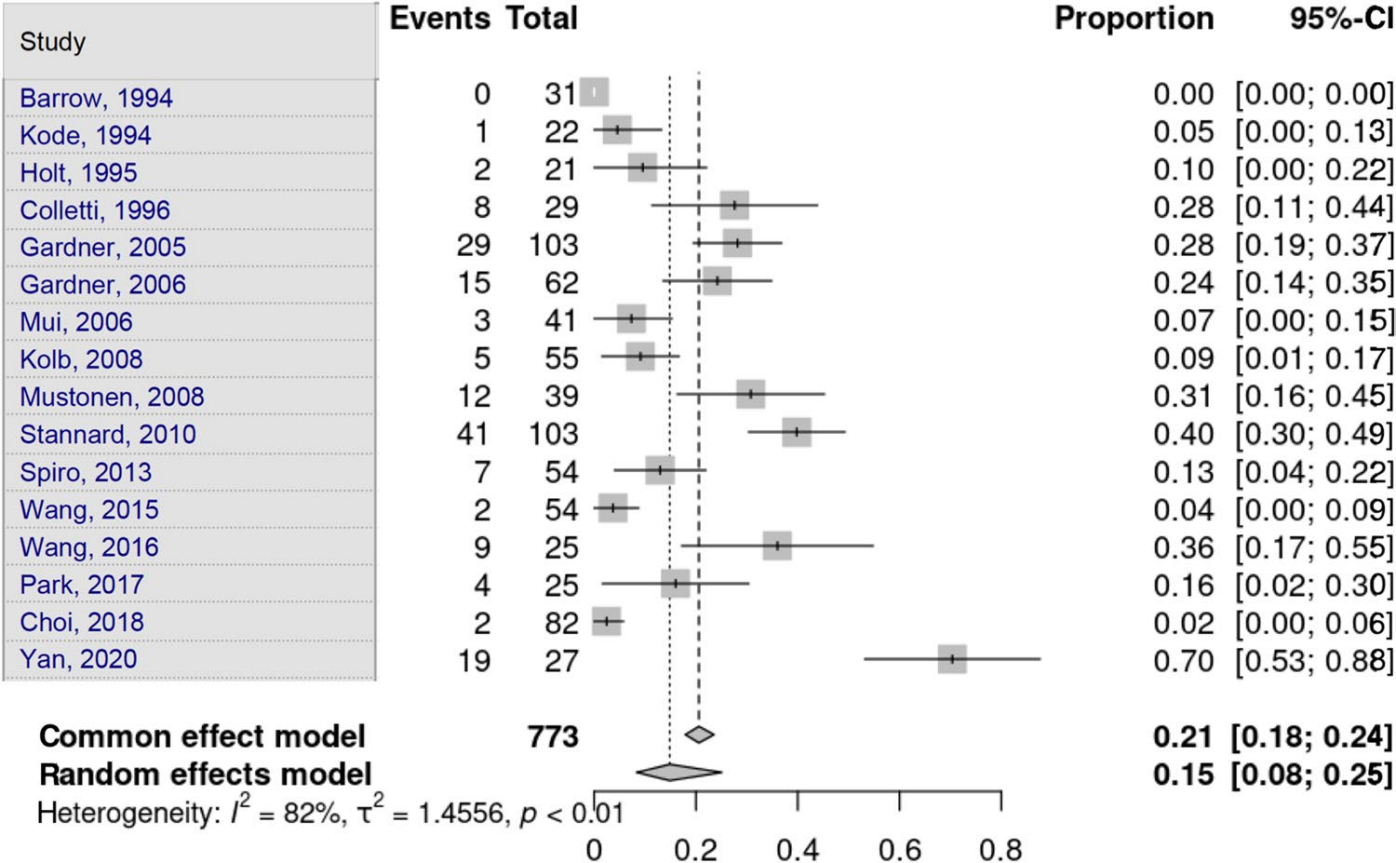
Posterior cruciate ligament lesion

PCL lesions were assessed by 16 studies. Heterogeneity in the study estimates was assessed using the *I*^2^ statistic (81.5%) and Cochran’s *Q* test (*p* < 0.0001), which showed heterogeneity. The overall proportion of 14.8% (95% CI 8.3–25.1) was independent of fracture type. This was a statistically significant result. Egger’s test and Begg’s test showed no small-study effects (*p* = 0.755 and *p* = 0.964, respectively) (Fig. 6).

Injuries to the posterolateral corner (PLC) and popliteus tendon were referred to in two studies [12, 17]. However, the population analyzed in Gardner et al. [17] included only Schatzker type II fractures, and it is likely to have been the same population as that analyzed in Gardner et al. study [12].

**Fig. 7:**
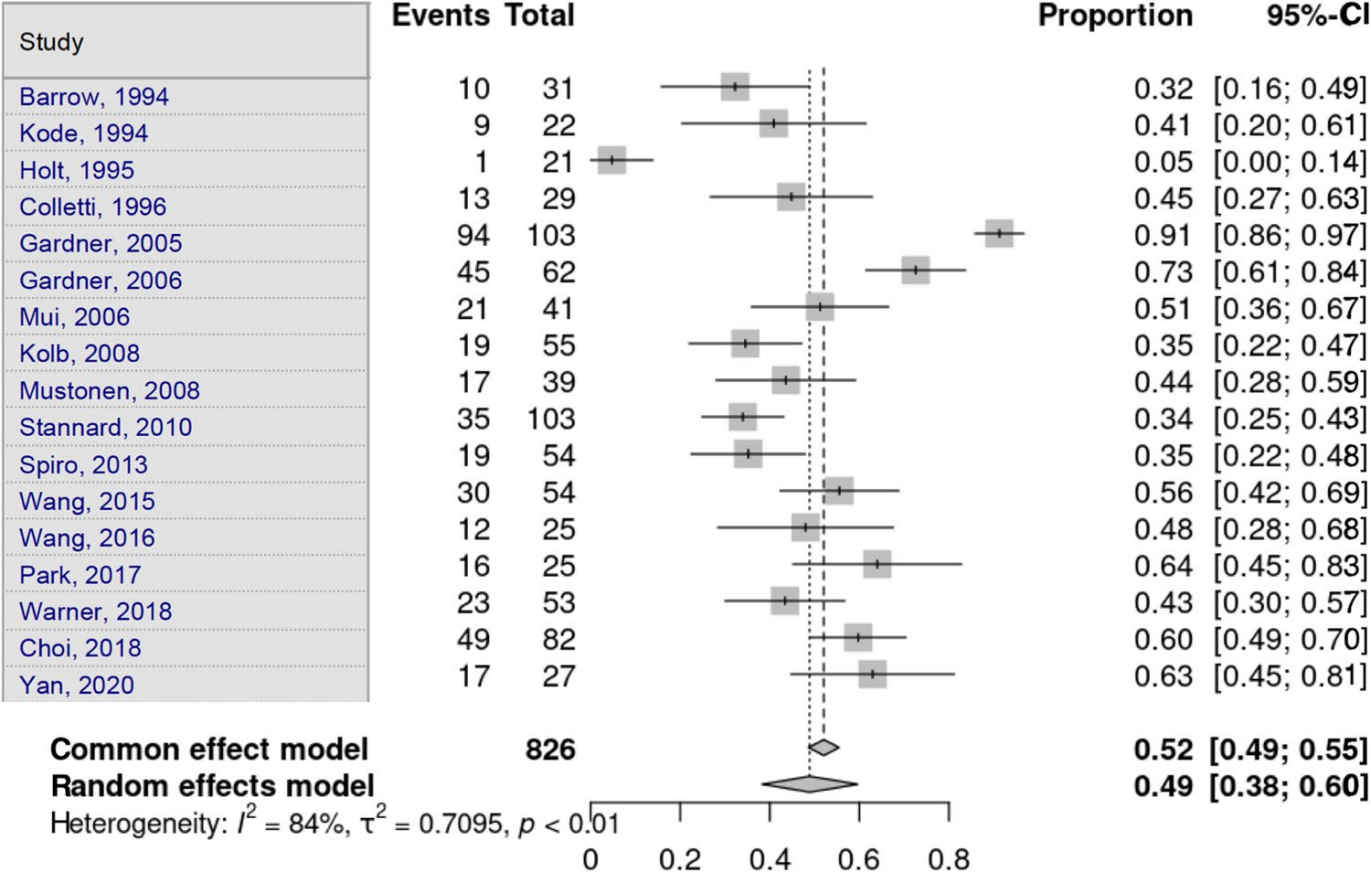
Lateral meniscus lesion

Choi et al. [16] identified only lateral meniscal lesions, and Yacoubian et al. [25] did not gather information on soft-tissue or meniscal lesions. Lateral meniscus lesions were assessed by 17 studies. Heterogeneity in the study estimates was assessed using the *I*^2^ statistic (83.9%) and Cochran’s *Q* test (*p* < 0.0001), which showed heterogeneity. The overall proportion of 48.9% (95% CI 38.28–59.62) was independent of fracture type, which was statistically significant. Egger’s test and Begg’s test showed no small-study effects (*p* = 0.109 and *p* = 0.588, respectively) (Fig. 7).

**Fig. 8:**
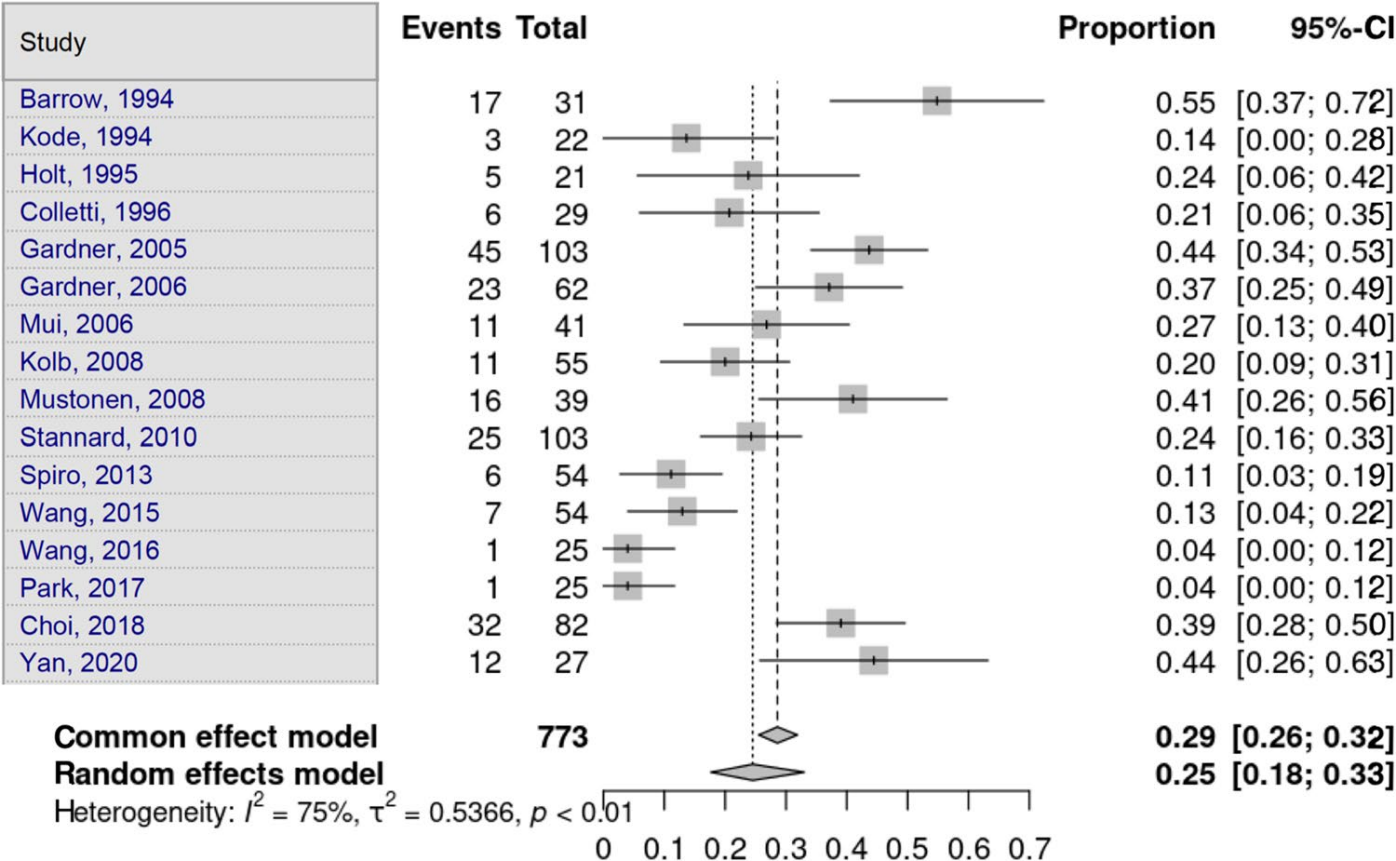
Medical meniscus lesion

Medial meniscus lesions were assessed by 16 studies. Heterogeneity in the study estimates was assessed using the *I*^2^ statistic (75%) and Cochran’s *Q* test (*p* < 0.0001), which showed heterogeneity. An overall proportion of 24.5% (95% CI 17.67–33.0) was independent of fracture type. This was a statistically significant result. Egger’s test and Begg’s test showed no small-study effects (*p* = 0.217 and *p* = 0.184, respectively) (Fig. 8).

### Association between individual soft-tissue lesions and type of fracture

A rank bi-serial correlation identified a significant relationship between the type of fracture according to Schatzker’s classification and an LCL lesion (*r*_rb_ = 0.209, *p* = 0.000, *n* = 331) or an ACL lesion (*r*_rb_ = 0.120, *p* = 0.029, *n* = 331). For both, there was a *weak* association according to Cohen’s test [31].

A rank bi-serial correlation identified no significant relationship between the type of fracture according to Schatzker’s classification and an MCL lesion (*r*_rb_ = − 0.104, *p* = 0.060, *n* = 331), PCL lesion (*r*_rb_ = − 0.029, *p* = 0.603, *n* = 331), medial meniscus lesion (*r*_rb_ = − 0.057, *p* = 0.348, *n* = 277), or lateral meniscus lesion (*r*_rb_ = − 0.068, *p* = 0.258, *n* = 277).

**Fig. 9:**
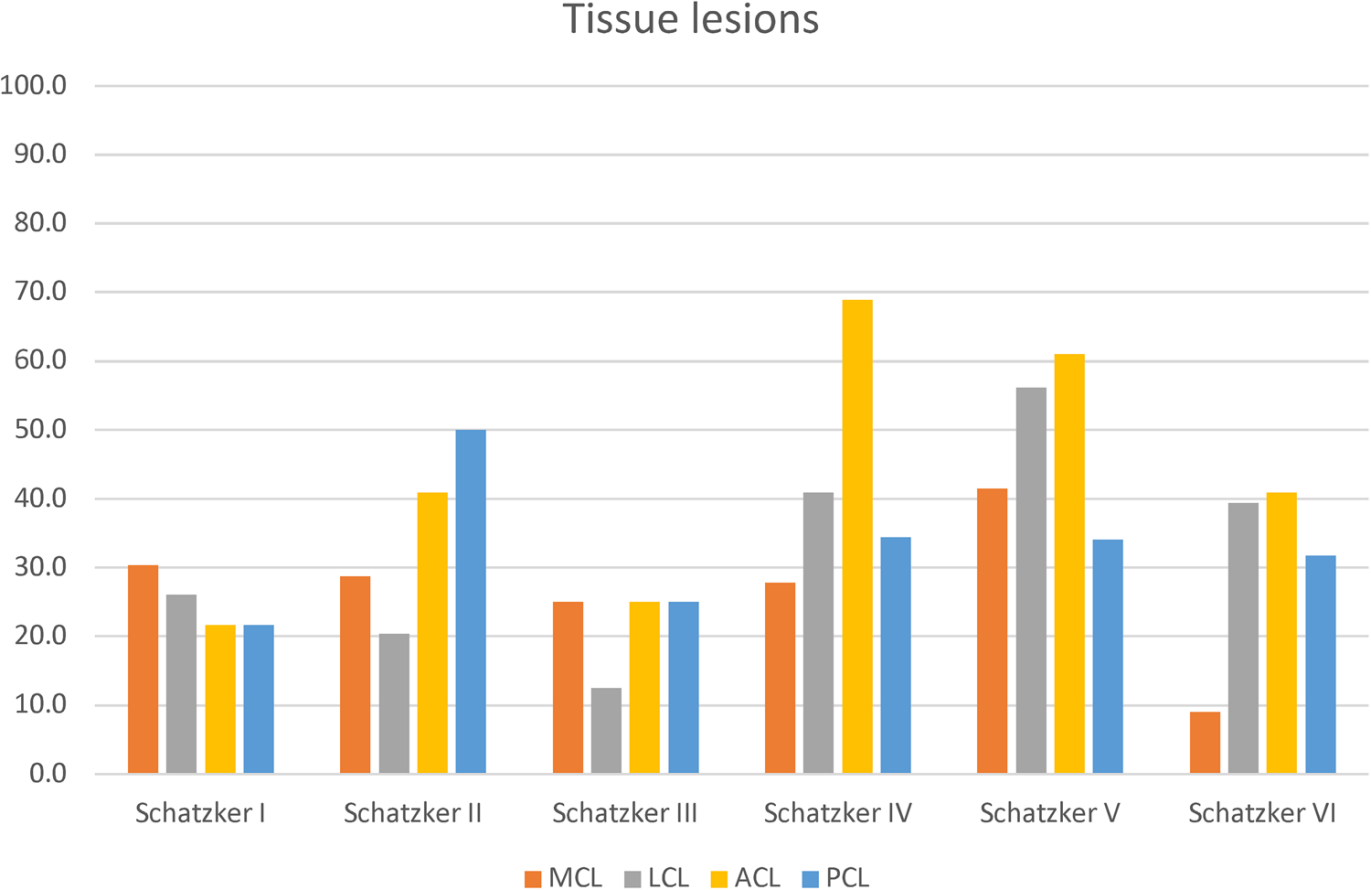
Frequency of ligamentous lesions in the tibial plateau fracture cohort. MCL medial collateral ligament, LCL lateral collateral ligament, ACL anterior cruciate ligament, PCL posterior cruciate ligament

**Fig. 10:**
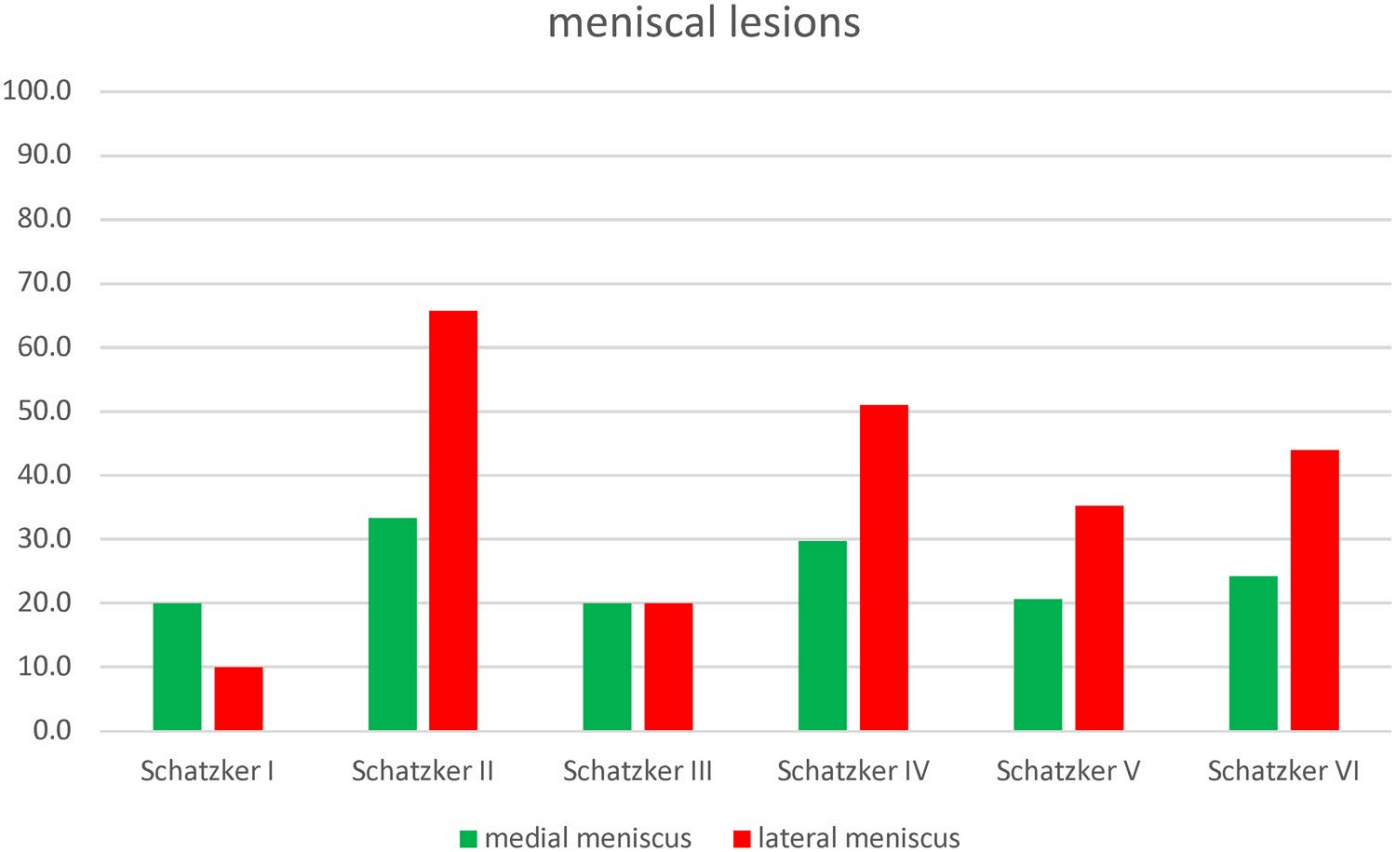
Frequency of meniscal lesions in the tibial plateau fracture cohort

Table 4 and Figs. 9 and 10 show the cumulative results of all the lesion subdivisions and the type of Schatzker fracture from 7 studies [11, 14, 15, 17, 22, 24, 26] and 6 studies [11, 14, 15, 17, 24, 26], respectively.

**Table 4:** Frequency of soft tissue injuries in the tibial plateau fracture cohort

Studies [6, 7, 11, 12, 19, 21, 23]	Schatzker I	Schatzker II	Schatzker III	Schatzker IV	Schatzker V	Schatzker VI
*n* =	23	132	8	61	41	66
MCL	7	38	2	17	17	6
LCL	6	27	1	25	23	26
ACL	5	54	2	42	25	27
PCL	5	66	2	21	14	21

Studies [6, 7, 11, 12, 21, 23]	Schatzker I	Schatzker II	Schatzker III	Schatzker IV	Schatzker V	Schatzker VI
*n* =	20	105	5	47	34	66
medial meniscus	4	35	1	14	7	16
lateral meniscus	2	69	1	24	12	29

*MCL* medial collateral ligament, *LCL* lateral collateral ligament, *ACL* anterior cruciate ligament, *PCL* posterior cruciate ligament

### Recommendations for preoperative MRI

The recommendations for and against preoperative MRI diagnostics are shown in Table 5 and are based on the conclusions of individual studies. Thirteen studies recommended additional MRI imaging if available [7, 8, 10–12, 14, 15, 17, 18, 20, 21, 25, 26], three studies found no further benefit [16, 22, 23], one was undecided [19], and one study’s recommendation was unclear [24]. No fracture type-specific recommendations were found. The responsible investigators were heterogeneous. In 5/18 studies [8, 14, 15, 20, 26], the investigators were blinded.

**Table 5:** MRI recommendation, type of investigators and if blinded

Study	MRI recommended?	Investigators/blinded
Barrow [22]	Yes	Orthopedic/blinded
Kode [26]	Yes	4 musculoskeletal radiologists and 2 orthopedic surgeons/n.a
Holt [10]	Yes	Experienced musculoskeletal radiologist/n.a
Colletti [11]	Yes	n.a
Yacoubian [20]	Yes, high energy	3 ortho (5–20 years)/random
Gardner [12]	Yes	Experienced musculoskeletal radiologist/n.a
Gardner [25]	Yes, if not available, CT with consideration of displacement	Experienced orthopaedic traumatologist/n.a
Mui [14]	Indecisive	Fellowship trained musculoskeletal radiologist/separate sessions, blinded?
Kolb [7]	Yes	1 trauma surgeon and 1 senior musculoskeletal radiologist (consensus decision)/n.a
Mustonen [15]	Yes, in high energy trauma (if = / > 3 mm articular step)	2 radiologists/blinded
Stannard [21]	Yes	Musculoskeletal radiologist/blinded
Spiro [8]	Yes	Musculoskeletal radiologist/blinded
Wang [17]	No	Musculoskeletal radiologist/n.a
Wang [23]	Yes	Radiologist/blinded
Park [16]	Yes	n.a
Warner [18]	No	Two orthopedics residents/n.a
Choi [24]	No	Musculoskeletal radiologist fellow/n.a
Yan [19]	Not clear	Musculoskeletal radiologist/n.a
Total	13 yes, 3 no, 1 indecisive, 1 not clear	

### Classification systems

Table 6 shows the preferred classification system used in the studies. The Schatzker classification [30] was applied in 17/18 studies, and the “Arbeitsgemeinschaft für Osteosynthesefragen Foundation/Orthopaedic Trauma Association” (AO/OTA) classification [32] was used in 11 studies. One study [24] used the Wahlquist classification [33], and another [15] used the quadrant classification [34]. Warner et al. [23] categorized Schatzker types I and II into one group and types III–VI into another group. Gardner et al. [17] analyzed only Schatzker type II fractures, and Yan [24] analyzed only Schatzker type IV fractures. The Schatzker and AO classification systems seem to be generally preferred, although they do not differentiate dislocation fractures, as suggested by Moore [35], for example.

**Table 6:** Classification

Study	Year	Schatzker I	Schatzker II	Schatzker III	Schatzker IV	Schatzker V	Schatzker VI	AO 41-B1	AO 41-B2	AO 41-B3	AO 41-C1	AO 41-C2	AO 41-C3
Barrow [22]	1994	2	13	2	4	2	8	–	–	–	–	–	–
Kode [26]	1994	2	9	4	2	2	3	–	–	–	–	–	–
Holt [10]	1995	1	16	10	4	8	1	41-B: 15			41-C: 4		
Colletti [11]	1996	3	14	3	3	3	3	–	–	–	–	–	–
Yacoubian [20]	2002	5	23	0	2	10	12	5	–	25	6	6	16
Gardner [12]	2005	3	62	–	17	5	14	3	0	79	17	11	3
Gardner [25]	2006	-	62	–	–	–	–	–	–	–	–	–	–
Mui [14]	2006	3	16	5	4	5	8	–	–	–	–	–	–
Kolb [7]	2008	4	50	1	–	–	–	–	–	–	–	–	–
Mustonen [15]	2008	–	–	–	–	–	–	13	17	7	1	1	–
Stannard [21]	2010	13	11	0	13	13	53	41-B: 37			41-C: 66		
Spiro [8]	2013	2	3	0	5	9	7	–	–	–	–	–	–
Wang [17]	2015	3	27	3	14	7	–	–	–	–	–	–	–
Wang [23]	2016	2	5	0	0	16	2	41-B1.1: 2	–	41-B3.1: 5	41-C1.2: 1; 41-C1.3: 2	–	41-C3.1: 13; 41-C3.3: 2
Park [16]	2017	-	15	10	–	–	–	41-B: 25	–	–	–	–	–
Warner [18]	2018	1	21	6	0	17	8	–	–	–	–	–	–
Choi [24]	2018	I–II: 55		–	IV–VI: 27			5	–	53	10	8	6
Yan [19]	2020	–	–	–	27	–	–	Total: 7; 41-B1.2: 3; 41-B1.3: 4	41-B2.3: 1	Total: 19; 41-B3.2: 2; 41-B3.3: 17	–	–	–

## Discussion

This review shows that at least one ligament or meniscal lesion is present in 93% of TPF cases. A weak association exists between an increasing frequency of LCL and ACL lesions and an increased type of fracture according to Schatzker’s classification [30]. The question of which cases are indicated for MRI cannot be fully answered by this review. However, the results suggest that MRI may be useful for high-energy trauma, fracture morphology with a dislocation mechanism, posterolateral fractures, and fractures with widening of the tibial plateau. [6, 20, 25, 26, 36].

Using conventional X-ray and CT as the primary diagnostic methods in TPF is standard. However, soft tissues cannot be assessed using these diagnostic tools. MRI is suitable for this purpose, as it provides accurate non-invasive assessment of knee pathology [37]. Several studies have demonstrated that as more tibial fracture displacement is shown on CT, the incidence of soft-tissue injury increases [7, 8, 16, 17, 38]. However, no threshold for articular depression or fracture displacement has yet been calculated to reliably predict or exclude soft-tissue injuries. In the absence of MRI, CT is certainly recommended, and various criteria based on the calculation of the articular depression, either of the lateral plateau or the tibial plateau itself, with or without calculation of the tibial plateau widening [7, 8, 16, 17, 22], allow calculation of the probability of soft-tissue lesions.

Various studies have investigated articular depressions and tibial plateau widening in TPFs to find an association with soft-tissue lesions. According to Kolb et al. [7], who performed an analysis of Schatzker types I–III, 1 mm of tibial plateau widening increases the likelihood of a lateral meniscal lesion by 40% and an LCL lesion by 32%. Spiro et al. [8] found in their analysis of Schatzker types I–VI that a tibial depression of 1 mm increased the likelihood of a lateral meniscal lesion in 15% of cases and an ACL lesion in 18%, and a soft-tissue lesion was present in 100% of the cases with a tibial plateau depression of 9 mm. Choi et al. [16] indicated that in the case of a lateral plateau depression of 5 mm, the sensitivity was 87% and the specificity was 80% for a lateral meniscus lesion in Schatzker Type I, II, IV, V and VI. Gardner et al. [17] calculated the likelihood of a type II meniscal lesion by relating lateral plateau depression to tibial plateau widening and found that the proportion of a > 6 mm depression and a > 5 mm widening was 83% for a lateral meniscal lesion and the proportions of a minimum 8 mm depression or widening were 53% and 78% for a medial meniscal lesion, respectively. Wang et al. [22] found that in cases of Schatzker types I–III with a lateral plateau depression of 5.6 mm with a widening of 7.4 mm found on conventional X-ray, one should suspect a lesion of the collateral or cruciate ligaments. In Schatzker types IV and V, this risk existed in cases of tibial plateau widening of 8.6 mm [22]. Likewise, Wang et al. made the same calculations specific to CT diagnostics. Overall, such calculations made on X-ray or CT remain only indicative of possible injury to internal knee structures. Crawford et al. [39] performed a systematic review comparing MRI to arthroscopy in the diagnosis of knee pathology. MRI appeared to be preferable to diagnostic arthroscopy in most patients because it is faster, avoids surgical risks, and identifies problems in each of the various tissues in and around the knee (ligaments, menisci, tendons, articular surface and bone), while keeping the investigation within acceptable times and costs. Mui et al. [19] found that CT can correctly detect a ligamentous lesion in 80% of the cases, but cannot reliably predict the status of the meniscal structures. The precise assessment of internal knee lesions requires MRI diagnosis [19, 39].

Identification of soft-tissue injuries is crucial for postoperative outcomes [4, 40]. A recent comparative study evaluating outcomes and functional status after knee fracture and knee fracture dislocation showed a higher incidence of ligamentous instability in knee fracture dislocation [5]. The incidence of reoperation due to persistent instability was also more frequent in this group. Furthermore, the incidence of OA increased significantly. At a mean follow-up of 17.5 months, both groups were similar regarding their pain on the Visual Analog Scale (VAS) and their standardized total short musculoskeletal function assessment (SMFA) scores. However, the groups were of different sizes and had a mean age of 49.8 (± 14.6) and 47.3 (± 14.3) years, respectively. Therefore, these results are more applicable to patients in this age segment. Snoeker et al. [2] performed a population study to estimate the risk of clinically diagnosed knee OA after different types of knee injuries in young adults with a mean age of 30 years. The risk of developing OA 19 years after sustaining a knee fracture was 6.6 times higher compared with the healthy population. Dislocation increased the risk by 6.7, ACL rupture by 19.6, and meniscal lesions by 10.5. A combination of cruciate ligament rupture and meniscal lesions showed a risk of 19.4. Because no distinction could be made between TPFs with and without soft-tissue lesions, it remains unclear which group is at higher risk for developing OA. An analysis of delayed multiligament knee reconstruction showed OA in 64.5% of the cases and that in 53.2% of the cases progression was most likely due to the initial injury [41]. TPFs with ligamentous lesions have a similar outcome to knee dislocations when treated in an acute setting [40]. Treatment not only of the fracture, but also of the soft-tissue lesions in an acute setting may reduce the risk of progression to OA. Missed soft-tissue lesions and the consequent delay in therapy may favor the development of OA. To prevent this, an accurate preoperative diagnosis by MRI is required.

The reviewed studies showed wide variation in the incidence rate of individual soft-tissue lesions, but most lacked precise information on grading and which lesions required surgical treatment. Mustonen et al. [20] documented 2/13 MCL lesions, 3/8 LCL lesions, 15/19 ACL lesions, and 9/12 PCL lesions in a patient population of 39 needing surgical reconstruction, but did not mention the decision-making process prior to surgery. Not all ligament lesions require surgery. Grades I–II MCL and LCL lesions, according to the Hughston classification [42, 43], and grades I–II PCL lesions, according to the Harner classification [44], can be treated conservatively. Grade III MCL, LCL, and PCL lesions are indicated for surgical therapy. It is unknown whether in the short- or long-term conservative therapy, in the presence of a Grade II MCL lesion, is superior to surgical therapy in the presence of a TPF. Whether a Grade II MCL lesion should be surgically addressed in the setting of an ACL rupture is still under debate. The Swedish National Knee Anterior Cruciate Ligament Registry [45] found that patients with an ACL rupture and a concomitant MCL lesion undergoing ACL reconstruction with MCL reconstruction or repair had reoperation rates similar to those with isolated ACL reconstruction. However, the patients who underwent ACL reconstruction without treatment for their MCL lesions showed an increased reoperation rate compared with those with isolated ACL rupture. Funchal et al. [46] presented their 2-year results of 112 patients with combined ACL rupture and MCL Grade II lesions with floating medial meniscus with similar results. Either isolated ACL or combined ACL with MCL reconstruction was performed. The MCL reconstruction group had a lower failure rate (3.0% vs. 29.0%), indicating that the surgical approach to MCL grade II lesions may result in a better outcome. However, the recent study by Lucidi et al. [47] found no advantage for surgical treatment of an MCL Grade II lesion in a combination injury with an ACL rupture. The same conclusion was reached by Halinen et al. [48], who found no differences, even in the presence of a grade III MCL lesion. This issue is still a matter of discussion. To date, there has been no comparative study with larger cohort groups examining surgical vs. conservative treatment of grade II MCL lesions in TPFs.

Conservative or surgical treatment of ACL lesions in the acute stage, regardless of grade, remains under discussion. However, clinical assessment of ligament stability alone may be insufficient, due to swelling and fractures, especially in nonoperative minimally displaced TPFs [6], but MRI can identify specific lesions well [9].

Injuries to the PLC are reported to be present in 16.0–28.0% of knee injuries and are frequently underdiagnosed [41–43]. They are overlooked particularly often in cases of PCL injuries, despite up to 70.0% of PCL injuries showing concomitant damage to the PLC [50, 52]. Untreated PLC injuries can lead to chronic pain and instability due to shifted biomechanics of the knee and, therefore, early development of OA [53]. They may also cause surgically isolated reconstructed cruciate ligaments to fail [54]. Only two studies reported PLC lesion incidences of 58.0% and 68.0% in combination with TPF, which is higher than the above-mentioned percentage [12, 17]. Gardner et al. published two studies that provided a global overview of 103 patients with PLCs with or without popliteus tendon lesions in 70 (68.0%) cases [12]. In 2006, the second study was published, which dealt only with Schatzker type II fractures and showed a PLC with or without popliteus tendon lesions in 36/62 (58.0%) of cases [17]. It may be the same cohort, and thus, the calculated average of 64.2% may be skewed. Warner et al. [23] described nine patients with LCL lesions (11.0%) who presented a complete tear of the popliteus in seven cases, a complete tear of the biceps femoris in four cases, and a complete tear of the popliteofibular ligament in four cases. Stannard et al. [26] reported 46 PLC lesions in 103 patients (44.6%). DeLee et al. [55] published a single-center study in which 12 cases (1.6%) suffered an isolated PLC injury and 22 cases (3.0%) a combined PLC injury in 735 knee ligament injuries. This difference in incidence indicates much uncertainty about the actual injury rate of this type. Similarly, no specific injuries to the posteromedial corner were described. Knowledge regarding lesions associated with TPFs in terms of frequency, significance, necessary therapy, and outcomes is still lacking.

The literature reviewed reports a frequency of 48.9% for lateral meniscus lesions and 24.5% for medial meniscus lesions (Table 3), which tend to occur in Schatzker types II, IV, V, and VI (Table 4), which is in line with reports using arthroscopy as a diagnostic tool [49–51].

In the presence of a longitudinal, radial, or root tear, surgery is indicated. Initial treatment leads to positive long-term outcomes [49–51]. Possible overtreatment [24, 56] or undiagnosed meniscal lesions [13] are reasons for using MRI to evaluate menisci in trauma cases. However, the retrospective study by Stahl et al. [56] relied on surgical reports, and all the patients who did not undergo surgical anterolateral arthrotomy were excluded from the study. The question of whether every meniscus lesion really needs to be treated also arises. Warner et al. [23] showed that medial meniscal lesions can heal without suturing, but did not specify what type of lesions were present. Elsoe et al. [38] found no significant difference in the Knee Injury and Osteoarthritis Outcome Score (KOOS) between the groups with (1 ACL lesion, 1 PCL lesion, 3 MCL lesions, 3 LCL lesions, 18 lateral, and 11 medial meniscal lesions) and without soft-tissue injuries in their patient population with TPFs, who were all treated without arthrotomy. It should be noted, however, that 7.0% of the patients were lost to follow-up, and 14.0% required a second procedure (2 total knee replacements and 6 arthroscopies). Both groups comprised participants over 50 years of age, and the follow-up period was short.

One can argue that the quality of MRI is limited by acute soft-tissue swelling or an external fixator due to a metal artifact. Nevertheless, MRI examinations in patients with non-ferromagnetic external fixators for traumatic knee dislocations can be safely performed under certain conditions and provide diagnostic-quality images [53–55, 57–59]. However, correct reading requires experience of this type of fracture [9].

The potential to overestimate soft-tissue injuries in MRI is high and requires extensive experience. Therefore, radiologists and surgeons should be familiar with how specific injury patterns influence management to achieve optimal outcomes [9].

The most commonly used systems for classifying TPFs were the Schatzker [30] and AO classifications [32]. One review indicated that these have “moderate” to “excellent” interobserver reliability [60]. With this discrepancy in reliability and given that no study could establish a correlation between different fracture types and soft-tissue lesions, the question arises as to whether another classification system would be more appropriate to analyze this relationship.

As presented in Table 5, 13/18 studies recommended the use of MRI as a preoperative diagnostic tool, especially in high-strain trauma cases [20, 25] and in young patients [6]. However, there is neither clear evidence nor conclusions as to which cases an MRI is indicated for, and this question cannot be clearly answered based on this review. The data collected by Warner et al. [23] suggest that obtaining a preoperative MRI to diagnose soft-tissue injuries in patients with a TPF may not alter surgical treatment or patient prognosis for mid-term outcomes, but this remains a hypothesis. The information obtained from MRI scans helps surgeons obtain a better idea of the damage and develop a plan [25]. Prior knowledge of these lesions could help surgeons better plan their surgical approach. However, whether this would also shorten the duration of surgery (and therefore reduce complication rates and hospital stays) and increase the quality of treatment and outcome (and, thus, have a socioeconomic impact) is a matter for future study. Information on the outcome of missed ligamentous lesions in TPF remains sparse in the literature, and few recent studies have examined the long-term outcomes (> 5 years) of conservative and surgical management of ligamentous structures associated with TPFs.

Accurate preoperative identification of all injured structures by MRI is useful. The current review shows that the literature does not allow a clear recommendation. However, from clinical practice experience and the available literature, the authors believe MRI is indicated in patients with high-energy trauma, fracture morphology with a dislocation mechanism, posterolateral fractures, and fractures with tibial plateau widening. However, this recommendation should be supported with further studies.

### Limitations

This review has some limitations. The review only considered studies using conventional X-ray, CT, and MRI. This review could not assess a correlation between soft-tissue lesions and fracture type, but reports the sum of soft-tissue injuries. The presence of high heterogeneity regarding patients’ mode of trauma, the classification used, and diversity in the design of the individual studies means that the data comparisons were of poor quality. Most studies have a level of evidence of type IV. Future studies should be more uniform in the assessment of the internal structures of the knee, trauma mechanisms and severity, and treated soft-tissue lesions.

## Conclusion

This review shows that at least one ligament or meniscal lesion is present in 93% of TPF cases. A weak association exists between an increasing frequency of LCL and ACL lesions and an increase in fracture type according to Schatzker’s classification. The indications for preoperative MRI are still a matter of discussion, and a standardized algorithm for MRI in TPF cases is still lacking. Nevertheless, preoperative MRI for the evaluation of soft-tissue injuries is an important diagnostic tool. More studies with higher levels of evidence are needed to find out in which particular cases MRI adds value.
